# Discontinuation of Levetiracetam and Valproic Acid Due to Adverse Effects in Early Post-traumatic Seizure Prophylaxis

**DOI:** 10.7759/cureus.47742

**Published:** 2023-10-26

**Authors:** Sami M Pathak, Robert Ziechmann, Jacob Menzer, Ava Hoeft, Philip Villanueva

**Affiliations:** 1 Neurosurgery, Temple University Hospital, Philadelphia, USA; 2 Neurological Surgery, Southern Illinois University School of Medicine, Springfield, USA

**Keywords:** early post-traumatic seizure, post-traumatic seizure prophylaxis, post-traumatic seizures, drug-induced hepatotoxicity, drug-induced thrombocytopenia, valproic acid, levetiracetam

## Abstract

Introduction: Levetiracetam (LEV) and valproic acid (VPA) are two anti-epileptic drugs (AEDs) routinely used for post-traumatic seizure (PTS) prophylaxis at our institution. In our practice, VPA is used for its beneficial effects on behavioral agitation and headaches, but it is also associated with abnormal liver function tests (LFTs). Both medications may be associated with thrombocytopenia. There is less literature comparing the adverse effect profiles and discontinuation rates of LEV and VPA in the context of PTS prophylaxis. We conducted a quality improvement (QI) analysis to determine the safety of LEV and VPA for traumatic brain injury (TBI) patients at our institution. In particular, our QI analysis involved calculating the rates of discontinuation or change of drug regimen due to the adverse effects.

Methods: Our QI analysis focused on patients treated for TBI at our institution during a six-year period. We recorded the AED used and if the AED was discontinued or switched due to thrombocytopenia, behavioral agitation, headaches, or elevated LFTs (including elevated aspartate aminotransferase or alanine aminotransferase values). We also recorded the incidence of early PTS, defined as seizures within seven days of the TBI.

Results: Our QI analysis included patients with a mean age of approximately 49 years with nearly 75% males. The mean Glasgow Coma Scale (GCS) score was 12.88, with 73.11% of patients having a mild GCS. The three leading injury mechanisms were fall, assault, and motor vehicle collision. The three leading types of TBI were traumatic subarachnoid hemorrhage, subdural hematoma, and cerebral contusion. Among patients with no prior history of seizures, we found an early PTS incidence of 7.28%. For patients administered LEV and VPA, 0.11% (1/898) and 3.85% (4/104) had the medication discontinued or changed because of thrombocytopenia (p < 0.001), respectively. For patients on LEV, 4.01% (36/898) and 1.78% (16/898) had the medication discontinued or changed because of behavioral agitation and headaches, respectively. For patients on VPA, 2.88% (3/104) had the medication discontinued or changed because of hepatotoxicity. In total, 5.90% versus 6.73% (p > 0.5) of patients on LEV and VPA, respectively, had their medication regimens changed due to the adverse effects.

Conclusions: The incidence of early PTS in our patients is within the range of what has been reported in the literature. The rate of discontinuation of LEV and VPA on account of adverse events is low in the context of PTS prophylaxis. Both medications had similar overall rates of discontinuation. VPA was discontinued more frequently than LEV due to thrombocytopenia, but discontinuation was not common in either case. LEV is associated with behavioral agitation and headaches, which makes VPA a desirable alternative for patients suffering from these symptoms.

## Introduction

Traumatic brain injury (TBI) is among the most frequent causes of death and disability in young individuals [1]. TBI occurs in roughly 1.6 million individuals in the United States each year [2]. Over 50,000 people die from TBI each year, and 80,000 suffer irreversible neurological disabilities [2].

Among the neurological sequelae of TBI are post-traumatic seizures (PTS), which are defined as one or more seizure events following TBI [3]. Early PTS (EPTS) is defined as a seizure within seven days of the TBI [4]. Early seizures after TBI have been shown to be linked to increased time in the intensive care unit, increased overall time in the hospital, and increased likelihood of discharge to a nursing facility [5,6]. EPTS has an estimated incidence of 2.1% to 16.9% [6,7]. EPTS occurrence predicts late PTS (LPTS), defined as seizures more than seven days following the TBI, and post-traumatic epilepsy (PTE) [6]. PTE describes recurrent seizure episodes after TBI [4] and may represent as much as 20% of epilepsy overall [6,8,9]. Further investigation is required to characterize the pathophysiology of PTE as TBI includes a broad categorization of injury mechanisms, each with unique effects on the central nervous system [8]. Given the association of EPTS with LPTS and PTE [6], as well as the prolonged hospitalization and intensive care unit time associated with early seizures [5,6], improving our understanding of EPTS and its treatment is imperative.

Several drugs have been used for the prevention of PTS, with older retrospective studies describing the effectiveness of phenytoin [10,11]. Temkin et al. [10] conducted a randomized, double-blind trial of phenytoin for the prevention of PTS and concluded that phenytoin (PHT) decreases the incidence of EPTS but not LPTS, indicating that PHT may act to suppress early seizures rather than serving as a true prophylaxis. Reducing early seizures is important because close to 25% of individuals with an EPTS will develop LPTS [12].

At many institutions, levetiracetam (LEV) became the standard of care for PTS prophylaxis due to its improved safety profile and equal effectiveness relative to PHT [13,14]. Xu et al. [13] performed a systematic review comparing the safety and efficacy of LEV vs. PHT in PTS and concluded that both drugs have similar efficacy in preventing PTS, but LEV has a more favorable safety profile. Bakr and Belli [14] performed a systematic review and found comparable incidence of late seizures between LEV and PHT, and long-term outcomes are improved with LEV. These reviews demonstrate a move away from PHT toward LEV for seizure prophylaxis due to the improved adverse effect profile and equal effectiveness of LEV relative to PHT [13,14].

Both LEV and valproic acid (VPA) are used regularly at our institution for PTS prophylaxis. VPA is associated with a beneficial effect on behavior and is routinely used for the treatment of psychiatric disorders [15-18], whereas LEV is associated with behavioral disturbances as an adverse effect [19]. Both drugs are associated with thrombocytopenia as an adverse effect [15,19,20]. VPA has a role in the management of headaches [15,21-23], whereas LEV is associated with headaches as an adverse effect [19]. An additional adverse effect of VPA is hepatotoxicity [15]. To characterize the safety profiles of these medications in PTS prophylaxis, we conducted a quality improvement (QI) study investigating the rates of discontinuation or change of drug regimen.

## Materials and methods

Study design

We performed a retrospective QI analysis in the Department of Neurosurgery, Division of Neurotrauma and Critical Care at Temple University Hospital in Philadelphia, Pennsylvania. Institutional review board approval is not required for QI projects, such as ours investigating patient safety. The data was pulled from the electronic medical record of patients with hospital admission for TBI anytime between January 2015 and June 2021 for a six-year total duration. We used a built-in feature of Epic (Epic Systems Corporation, Madison, Wisconsin) to select the relevant patients using the diagnoses described in our inclusion criteria as captured in the current procedural terminology codes.

Inclusion and exclusion criteria

The inclusion criteria required the patient’s hospital encounter to be for a new TBI. We included all adult patients with the following types of TBI: traumatic subarachnoid hemorrhage (TSAH), subdural hematoma (SDH), cerebral contusion (CC), intracerebral hemorrhage (ICH), and epidural hematoma (EDH). Patients readmitted for subsequent hospital encounters related to their original trauma and patients on VPA for psychiatric diagnoses rather than PTS prophylaxis were excluded from the QI analysis. Patients with aneurysmal subarachnoid hemorrhage and spontaneous ICH as well as patients with SDH secondary to elective intracranial surgery were also excluded. For our calculation of EPTS incidence, we excluded patients with a prior history of seizures.

Safety and quality assessment measures

We reviewed the electronic medical record for each included encounter. We recorded the patient’s age, sex category (male or female), and Glasgow Coma Scale (GCS) score at presentation for TBI. For the GCS score, we defined mild GCS as 13-15, moderate as 9-12, and severe as 3-8. We recorded the mechanism of injury, including motorcycle collision, motor vehicle collision, all-terrain vehicle collision, assault, fall, gunshot wound, or other. We recorded the type of TBI as defined above (TSAH, SDH, CC, ICH, or EDH). For each encounter, we recorded if EPTS (defined as a clinical or electrographic seizure) occurred. We also recorded whether each patient received an order for LEV or VPA and if there was a switch from LEV to VPA or vice versa. The primary outcome of our study was the rate of drug discontinuation or drug regimen change due to various adverse effects. We recorded if either drug was discontinued due to thrombocytopenia. This was determined by searching the progress notes on the electronic medical record (EMR) for the hospital encounter corresponding to this admission. We recorded if LEV was switched to VPA (or if VPA was added to LEV) due to headaches or behavioral agitation, which we determined by searching the progress notes in the EMR. We recorded if VPA was discontinued due to hepatotoxicity, which we determined by searching the progress notes in the EMR.

Statistical analysis

We performed two chi-square tests for our statistical analysis, each with one degree of freedom. The first compared the rate of drug discontinuation due to thrombocytopenia between the number of hospital encounters with patients prescribed LEV versus encounters with patients prescribed VPA, and the second test compared the rate of drug regimen change due to all adverse effects combined for each medication (e.g., thrombocytopenia, behavioral agitation, and headaches for LEV versus thrombocytopenia and hepatotoxicity for VPA). All statistical analysis was performed in MATLAB R2021a (MathWorks, Natick, Massachusetts).

## Results

**Figure 1:**
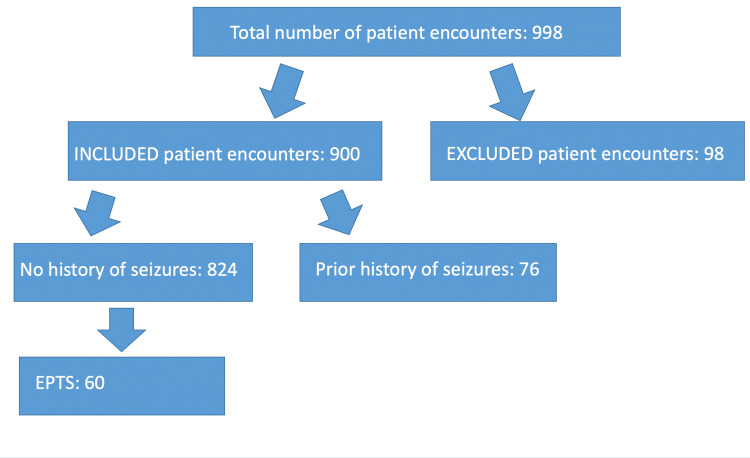
Flow chart of inclusion and seizure status We include a flow chart of the inclusion and seizure status as described in Table 1. We describe the total number of patient encounters, the excluded patients, the included patients, seizure history status, and the incidence of EPTS. EPTS: Early post-traumatic seizures.

Table 1 and Figure 1 (a flow chart) both demonstrate the inclusion and EPTS status. There were 998 patient encounters in our report, of which 98 encounters were excluded because they involved a hospital admission not related to a TBI or not corresponding to a new TBI. A total of 900 patient encounters were included in the study, of which 76 of the included patients had a prior history of seizures, and 824 had no known prior history of seizures. Of the patients with no prior history of seizures, EPTS was observed in 7.28% (60/824).

**Table 1:** Inclusion and seizure status We describe the total number of patient encounters, the excluded patients, the included patients, seizure history status, and the incidence of EPTS. *We calculate the incidence of EPTS based on the total number of patients without a prior history of seizures. EPTS: Early post-traumatic seizures.

Category	Number of patients
Total patient encounters	998
Excluded	98
Included	900
Prior history of seizures	76
No prior history of seizures	824
EPTS*	60/824 (7.28%)

Table 2 demonstrates the demographic data of our QI study, which helps provide the clinical context for our patient population. Among the 900 included patients, the mean age was 49.31 years, with 74.56% and 25.44% male and female, respectively. The mean GCS was 12.88, with 73.11%, 13.56%, and 13.33% of patients having mild, moderate, and severe GCS scores, respectively. Table 3 represents the traumatic injury mechanism. The most common injury mechanism was fall at 47.22%. If there was no clear mechanism in the EMR, we recorded multiple types of traumatic injury mechanisms (e.g., falls and assaults) for certain patients. Table 4 represents the type of TBI sustained. Many patients had multiple TBI types reported in their radiographic studies. TSAH was the most common type of TBI at 77.67%, and EDH was the least common at 15.56%.

**Table 2:** Demographic data for the quality improvement study We report the mean age, sex category, mean GCS, and GCS categorization. Mild, moderate, and severe GCS represent GCS scores of 13-15, 9-12, and 3-8, respectively. The GCS score and GCS categorization refer to the value at the patient’s initial presentation for the TBI. GCS: Glasgow Coma Scale; TBI: Traumatic brain injury.

Mean age (years)	Male	Female	Mean GCS	Mild GCS	Moderate GCS	Severe GCS
49.31	671 (74.56%)	229 (25.44%)	12.88	658 (73.11%)	122 (13.56%)	120 (13.33%)

**Table 3:** Traumatic injury mechanism We report the percentage of patients with each traumatic injury mechanism. MCC: Motorcycle collision; MVC: Motor vehicle collision; ATV: All-terrain vehicle; GSW: Gunshot wound.

MCC	MVC	ATV	Assault	Fall	GSW	Other
48 (5.33%)	88 (9.78%)	8 (0.89%)	178 (19.78%)	425 (47.22%)	54 (6%)	145 (16.11%)

**Table 4:** Type of traumatic brain injury We report the type of traumatic brain injury sustained. TSAH: Traumatic subarachnoid hemorrhage; SDH: Subdural hematoma; CC: Cerebral contusion; ICH: Intracerebral hemorrhage; EDH: Epidural hematoma.

TSAH	SDH	CC	ICH	EDH
699 (77.67%)	502 (55.78%)	397 (44.11%)	368 (40.89%)	140 (15.56%)

Table 5 demonstrates the medication status. A total of 796 patients were given LEV only for PTS prophylaxis during admission, and two patients were given VPA only. Sixty-five patients were switched from LEV to VPA, two switched from VPA to LEV, and six switched from LEV to VPA and back to LEV (LEV and VPA both switch). Twenty-nine patients were on LEV and had VPA added to their regimen (LEV and VPA no switch). In total, 898 patients received LEV, and 104 patients received VPA.

**Table 5:** Medication status We recorded if the patient received the following: LEV only, VPA only, LEV initially and then switched to VPA (LEV to VPA), VPA initially and then switched to LEV (VPA to LEV), LEV to VPA and then switched back to LEV, both LEV and VPA (no change between the two medications), the total number of patients on LEV, and the total number of patients on VPA. LEV: Levetiracetam; VPA: Valproic acid.

Medication status	Number of patients
LEV only	796
VPA only	2
LEV to VPA	65
VPA to LEV	2
LEV to VPA and VPA to LEV	6
LEV and VPA (no switch)	29
Total LEV	898
Total VPA	104

Table 6 demonstrates the rates of drug discontinuation or drug regimen change. This rate is defined as the total number of patients with drug discontinuation or drug regimen change for that particular adverse effect divided by the total number of patients receiving the drug. LEV was discontinued due to thrombocytopenia in 0.11% (1/898) of patients receiving LEV. VPA was discontinued due to thrombocytopenia in 3.85% (4/104) of patients receiving VPA. A chi-square test was performed to compare the incidence of drug discontinuation due to thrombocytopenia. With one degree of freedom, our chi-square test results in a p-value of less than 0.001. With a significance level of 0.05, this result is statistically significant.

**Table 6:** Rates of drug discontinuation or drug regimen change for each adverse effect A p-value comparing the rates of discontinuation or drug regimen change for levetiracetam and valproic acid is demonstrated for thrombocytopenia and all adverse effects combined. LEV: Levetiracetam; VPA: Valproic acid; N/A: Not applicable; LFT: Liver function test.

Cause of medication change	LEV (898 total)	VPA (104 total)	P-value
Thrombocytopenia	1 (0.11%)	4 (3.85%)	<0.001
Behavioral agitation	36 (4.01%)	N/A	N/A
Headaches	16 (1.78%)	N/A	N/A
Elevated LFT	N/A	3 (2.88%)	N/A
All adverse effects combined	53 (5.90%)	7 (6.73%)	>0.5

Of the patients receiving LEV, 4.01% (36/898) were given VPA as an add-on medication to LEV or switched to VPA because of behavioral agitation. Of the patients receiving LEV, 1.78% (16/898) were given VPA as an add-on medication to LEV or switched to VPA because of headaches. Of the patients receiving VPA, 2.88% (3/104) were discontinued from VPA due to elevated liver function tests (LFTs). Overall, 5.90% (53/898) of patients receiving LEV had the medication discontinued or were started on VPA in addition to LEV due to adverse effects of thrombocytopenia, behavioral agitation, or headaches. Overall, 6.73% (7/104) of the patients receiving VPA had VPA discontinued due to thrombocytopenia or elevated LFTs. A chi-square test comparing the LEV and VPA groups yielded a p-value of greater than 0.5, indicating no statistically significant difference between the groups.

## Discussion

The use of antiepileptics to prevent early PTS is predicated on reducing the harm of early seizure while assuming a low risk of adverse effects from the medication. We found an EPTS incidence of 7.28%, which is within the range of those reported in the literature [6,7,24,25] of up to 16.9%.

Our results demonstrate that despite the adverse effect profiles associated with LEV and VPA use, the rate of discontinuation due to adverse events is low, with overall rates of medication regimen change of 5.90% and 6.73% for LEV and VPA, respectively. The difference in rates of discontinuation for thrombocytopenia between LEV and VPA was statistically significant, but both rates were small. The rates of LEV discontinuation or medication change for agitation and headaches were slightly larger than those for thrombocytopenia, with 4.01% and 1.78% of patients on LEV requiring medication regimen change for these adverse effects, respectively.

Although many patients on these medications experience a decrease in platelets (PLT), the cause of this change may be multifactorial. For example, many patients experience acute coagulopathy due to their traumatic injuries [26]. Measuring the PLT count alone does not necessarily indicate thrombocytopenia due to the medication. Recording whether or not the medication was discontinued discloses more information regarding the effect of the medication on PLT count.

Buoli et al. [20] reviewed the risk of thrombocytopenia with VPA use; the authors found that in studies with a sample size of more than 150 subjects, 12%-18% of the subjects receiving VPA therapy developed thrombocytopenia. Risk factors for thrombocytopenia in their study included advanced age, female gender, and high doses of VPA [20]. The article studied VPA use in general, including its use in psychiatry and epilepsy [20]. Our study is different in a way that we examined the population of patients receiving PTS prophylaxis. Our result of 3.85% discontinuation in the VPA group is lower than the rate of thrombocytopenia published by Buoli et al. [20]. This may be explained by the shorter time course of recording data for PTS prophylaxis compared to those with other indications for VPA use. According to Howard et al. [19], thrombocytopenia is classified as a common (<10%, >1%) side effect of LEV. Like VPA, our result of 0.11% discontinuation in the LEV group is lower than the estimate. It is often difficult to characterize whether a decrease in PLT is due to medication effects or other causes, and we expect the rate of discontinuation of the medication due to thrombocytopenia to be less than the actual incidence of thrombocytopenia. Sahaya et al. [27] performed a retrospective study and found that in 758 patients on LEV, 29 had associated thrombocytopenia, but only one of these patients had thrombocytopenia that was likely associated with LEV. Our rate of LEV discontinuation due to thrombocytopenia (0.11%) is close to their rate of 0.13% (1/758) of likely LEV-induced thrombocytopenia [27]. Not all decreases in PLT due to the medication are of sufficient clinical significance to discontinue the medication. Our results demonstrate that thrombocytopenia is likely more of a concern in patients receiving VPA than LEV.

According to Howard et al. [19], behavioral disturbances such as emotional lability, irritability, agitation, hostility/aggression, and personality disturbances are common adverse effects of LEV, with a rate between 1% and 10%. In our QI analysis, 4.01% of patients receiving LEV required the addition of VPA or replacement with VPA due to behavioral agitation, which is consistent with the reported rate of Howard et al. [19]. Howard et al. [19] described headache as a very common adverse effect of LEV, with an estimated rate of more than 10%. About 1.78% of patients in our QI analysis receiving LEV were switched to VPA or had VPA added due to headaches. Powell-Jackson et al. [28] reviewed hepatotoxicity in VPA usage, reporting an estimated incidence of abnormal serum aminotransferase levels of 0%-44%, with an overall incidence of 11% in the total number of patients recorded across the trials. About 2.88% of the patients receiving VPA in our QI analysis required drug discontinuation due to abnormal LFTs. As we recorded the number of instances in which the medication regimen was changed rather than the absolute number of occurrences of the adverse effect, we expect that our rates of drug regimen change do not necessarily align with the rates of occurrence of the adverse effects in the literature. A report by Sussman and McLain [29] from 1979 described nine patients hospitalized for epilepsy who were treated with VPA and developed liver function abnormalities, with four of these patients developing low platelet count. VPA was discontinued in only three of these patients, with lab values returning to normal when the dosage was decreased or the medication was discontinued [29]. The relatively low rate of medication discontinuation demonstrates that not all changes in lab values are of clinical significance to necessitate medication stoppage, and decreased medication dosage is often sufficient [30]. Regardless, these demonstrate proof of principle that the common anti-epileptic drugs (AEDs) used for PTS prophylaxis at our institution each have distinct adverse effect profiles and should continue to be selected based on the particular patient.

Limitations

The main limitation is that our QI analysis is retrospective and observational in nature. In addition, many of the patients in our analysis had a prior history of seizures before the TBI. Many of these patients had received other AEDs in the past or were already on AED regimens at the time of presentation for TBI. These patients may have already been experiencing adverse effects, which limits our analysis of the specific population of patients receiving EPTS prophylaxis. While we excluded patients with a prior history of seizures from the calculation of EPTS incidence, it is possible that several of the included patients had a prior history of seizures that we could not encounter in the documentation. In addition, we focused on adverse effects experienced during the initial hospital admission related to TBI rather than all subsequent hospital encounters. It is possible that more changes were made to the AED regimen due to adverse effects after the initial TBI encounter.

## Conclusions

Overall, our study demonstrates that LEV and VPA had relatively low rates of discontinuation for patients receiving PTS prophylaxis. Thrombocytopenia is an important adverse effect to monitor for patients receiving either medication, but clinically harmful thrombocytopenia leading to drug discontinuation is expected to be rare. Rates of thrombocytopenia are expected to be higher for patients receiving VPA, and therefore drug discontinuation due to thrombocytopenia is expected to be higher for VPA patients. Behavioral agitation and headaches are adverse effects to monitor for LEV, and elevated LFTs may be observed with VPA. Both medications have similar overall rates of drug regimen change for their adverse effect profiles, and both medications are safe for use in PTS prophylaxis. It has been our practice at our institution that we consider VPA for patients with behavioral agitation and headaches, and this appears to be a reasonable choice based on our safety data.
